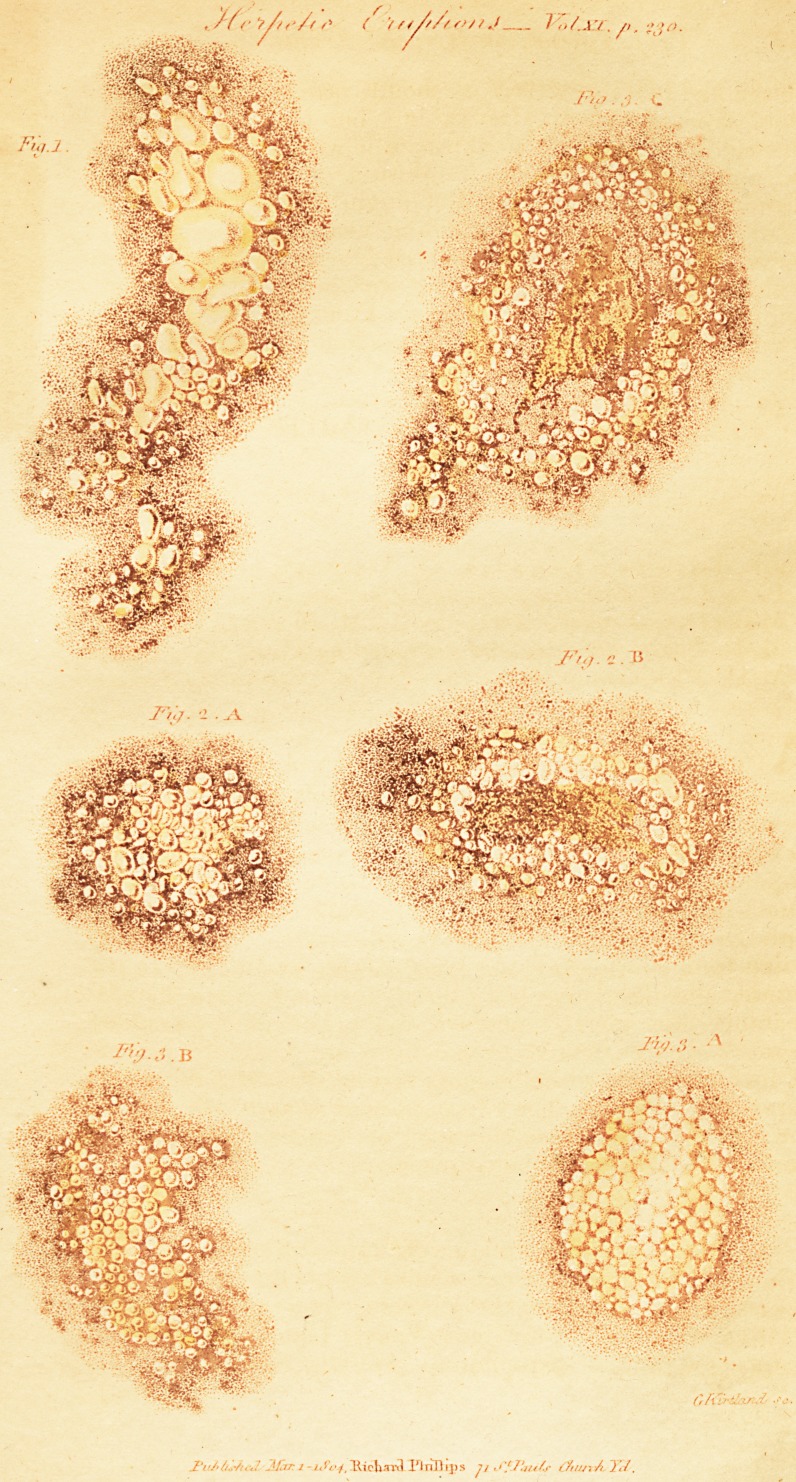# Prof. Tilesius's Remarks on Herpetic Eruptions, Part I. Translated from the Paradoxien of Dr. F. H. Martens, Leipsig, 1802

**Published:** 1804-03-01

**Authors:** T. M. Winterbottom, Thos. Bateman

**Affiliations:** South Shields; Physician to the Public Dispensary in Carey Street


Prof. Tilesius's Remarks on Herpetic Eruptions, Part I. *Translated from the Paradoxien of* Dr. F. H. Martens, *Leipsig,* 1802; *by* T. M. Winterbottom, M.D. *South Shields; and abridged by* Thos. Bateman, M.D. *Physician to the Public Dispensary in Carey Street.*

[ With an Engraving. ]

After having collected the histories of cutaneous diseases, which occurred in his practice during a period of several years, Dr. Tilesius observed that cases of the *herpetic* eruptions were the most numerous; and he was induced to publish his observations in consequence of the confusion which prevails among writers on this subject. He accords with Dr. Willan in the remark, that this confusion has arisen from considering the same disease as different in its nature, and designing it by different names, when its appearance was merely modified by, 1. Difference of situation. 2. By the degrees of violence; or, 3. By the period of its duration; and he adduces several examples to corroborate this remark. The two important sections which follow, treat of the generic character of herpes, and the division and description of the species.

*Generic Character of Herpes.*

The true *herpetic* eruption has hitherto, even by late writers, had various generic names assigned to it, and has been arranged under all the following genera, viz. Lichen, (Willan, Plenck.) Impetigo, (Vogel, Lommius, Plenck.) — Psora, (Lommius.) Strophulus, (Willan, Strophulus, Volaticus.)

Herpetic Eruptions — Vol. XI. p. 230.

G. Kirtland sc.

Published Mar. 1-1804, Richard Phillips 71 St. Pauls Church Yd.

Gaticus. And even under Scabies, (Ludwig, and others, who merely looked to the cure.) To the causes which have given occasion to this confusion, so well pointed out by Willan, I shall add, that the basis, or broad, inflamed area of this eruption, has never been examined with the microscope; otherwise it would have been seen, that the small pimpled herpes presents to the armed eye the same form as the miliary eruption and pemphigus do to the naked eye; and that the general character of herpes is a circular, oval, or even irregular, itchy, inflamed, and somewhat elevated basis, upon which the congregated, and often confluent pimples (papulæ gregales) spring up. Sauvages (Cl. i. Ord. 2) is tolerably accurate. Some of the moist kinds of herpes acquire small, yellow crusts, produced by the saline, glutinous fluid oozing from the pimples when burst; these project like solitary rough points from the area, and produce the characteristic rough surface. It was probably only these incrusted points which Sauvages has termed "crustaceas"; for, except these, no crusts appear upon herpetic eruptions; only branny scales, in which the area, or inflamed basis, commonly desquamates. Sometimes the basis appears wrinkled, when the epidermis has been so much stretched and elevated during the inflammatory period, that subsequently, when it collapses, it lies in long folds, until it be sufficiently dry to exfoliate. Moreover, the acute sense of burning and itching, which continues even after the period of inflammation, is characteristic of this genus. Sauvages says very justly, "Papulæ pejores, corrosivæ, congestæ, aream circularem constituunt, acutè pungentem, valdè pruriginosam." Finally, all herpetic eruptions terminate in a regular desquamation.

It must be observed, that the desquammation of the cuticle is not affected in all diseases in precisely the same manner. It is sometimes separated in the form of scales, sometimes of bran, sometimes of meal or farina, and in some cases in that of foliola or flakes; always in a determinate form, according to the nature of the disease. Hence the importance of microscopical observations, in order to ascertain with accuracy the nature of the disease. Herpes and scabies agree in this, that their pimples break, and fall off in *branny* frustula epidermidis. The *area* of herpes however distinguishes it from itch, in which the pustules are solitary, and spread over every part.

Do pimples or papulæ, which Plenck, Sauvages, and other good Nosologists include in the generic character of herpes, and even consider as a principal appearance, exist in all herpetic eruptions? This is a question which Sennertus and Cullen (Phlyctenæ vel ulcuscula plurima, gregalia, serpentia, dysepuleta,) might have answered, if they had perused their works. Agreeably to my own observations, I must answer it in the negative. For I have frequently seen a real herpes, without papulæ, that is, a local eruption of small congregated vesicles (phlyctenæ,) placed upon a somewhat elevated, broad, inflamed basis. This eruption is figured in the plate, (fig. 1) and described under the name of bladder herpes (blasenflechte). It is much larger than the miliary herpes (hirseflechte) which has been described by Sennertus as vesicular, "Constat ex hydatidibus, seu vesiculis, granum milii æquantibus;" and hence its vesicles can be less easily mistaken than those of the miliaria, or considered as papulæ or pimples. Nosologists, however, have given very different definitions of papulæ. Sauvages has classed them with pustulæ, phlyctenæ, and vari, among the rashes, (efflorescentiæ). Plenck says, "papulæ are small hard tumours, which either disperse, or discharge out of their apices a fluid, and then scale away. They differ from pustules in not suppurating, and from vesicles in containing no watery fluid. They appear to be seated in the glands of the skin." Willan understands by papulæ, a very small and pointed elevation of the epidermis, with an inflamed basis, which contains no fluid, and does not suppurate, whose continuance is uncertain, and which mostly terminates by a branny desquamation. All these definitions, however, are ambiguous, because they rest upon no anatomico-pathological examination.

The reason why phlyctis or vesicula is transparent, and on the contrary papula is hard and opake, is to be deduced from the organs by which these eruptions are formed. The first is merely a separation or elevation of the cuticle from the rete mucosum, and is occasioned by an effusion and collection of acrid lymph. In the latter, the acrid lymph, stagnating in the skin and adipose membrane, imitates the cutaneous glands, and forces them, covered with the rete mucosum, upwards to the surface. Consequently a phlyctis is a lymphatic elevation of the transparent epidermis alone; but a pimple (papula) is a slighter elevation of the epidermis, together with the rete mucosum. To be convinced of the justness of this explanation it is only necessary to open a phlyctis, in herpes vesiculosus, erysipelas, or zona, and compare it by means of a microscope, or even by the feel of the fingers, with a papula laid open in a patient affected with with itch, with lichen, or who has gruta, vari, or fonthi on the face.

Both of these kinds of eruption appear among the examples of herpes, but do not constitute its character. Independently of them, the flat, elevated, and broad area beforementioned, upon which the papulæ or vesiculæ of herpes are collected, suffice to characterize the genus herpes; for there is no other genus among the various eruptions in which this peculiar appearance is observable. Hence herpes is most easily distinguished from all other eruptions.

Herpes invariably retains its characteristic distinction, it seldom mixes or is complicated with other eruptions, and never changes into other genera. "Ex acri veneno degenerante herpeticum oriri, non minus, quam pomorum e semine prunos expectare, absurdum videtur." Frank de Cur. Hom. Morbis.) What Wichman says of eruptive poisons in general, is particularly applicable to herpes. "I have no idea of the degeneration of an exanthematous poison or virus; it may alter in some respects, assume a different form, and produce different effects, according as it attacks this or that part of the body; but it can no more degenerate or change into another kind, than a vegetable. It always continues to be a modification of the same virus, and requires only a little diagnostic skill to discover it. Syphilis affords a particular illustration; if it be not totally eradicated, it may perhaps be deposited upon a glandular part, produce hardness and congestion there, but not scrophula; and it will yield to no other than to its specific remedy. Such is the case with herpes, itch, &c. which change into each other as little as measles change into small-pox, or as the cow-pock changes into variola, and vice versâ. But one virus may be complicated with another kind, and so on."

Whether the complication of herpes with other kinds of virus be a rare occurrence or not, we are certain that it has been combined with erysipelas, and this *mixed* eruption has been called by Poupart, dartre erysipelateuse. This author distinguishes it from the other species, by its larger and more extensive inflamed base (area); and by the pimples or rather vesicles (bläschen), from which there oozes a thick, glutinous, saline, and almost purulent fluid, and which appear at first close and congregated in the midst of the area, and accompanied with great heat and violent itching.

A similar combination takes place in the venereal herpes, which generally appears on the upper and inner parts of the the thigh, the scrotum, and inguinal regions; in women, on the pudenda, mons veneris, papilla of the breasts. It is distinguished by very small, dry, reddish-yellow papula, which spring up upon a bluish red, inflamed base, (area) and are sometimes circumscribed by a sharp edge. Those who are affected with this eruption, have commonly been liable to herpes, before they were attacked by lues; and though the herpes has been somewhat changed by the accession of the syphilitic virus, it cannot be cured without mercury. Herpetic patients are no more secure from the effects of venereal virus, than patients affected with the itch. But it is remarkable, that herpes, in every possible mixture of lues, &c.) still retains its generic character, the area, and branny desquamation.

Many good practitioners seem to know this distinctive character; although it is often lost sight of, by making an unnecessary distinction between lichen and herpes. Wichman seems to have distinguished the herpetic pimple very well from other isolated pimples, by contrasting the crusta lactea and serpiginosa; but though he seems to comprehend it, he has not given the characteristic of herpes. He considers the more extensive basis of the inflammation, (or area,) the more violent itching, and the more intense redness, as also the congregated papulæ miliares of the one, opposed to the isolated ulcers, and pale red margins of the other, as affording a generic distinction. But it is the red spot, basis, or area, which distinguishes herpes from all other eruptions. Its progress is in this manner: Upon this red spot, which rises more or less above the surface, a number of pimples or vesicles appear closely crouded together, and which, upon the slightest touch on their summits, feel as if pricked with needles; at this time the itchy irritation has reached its acme, and the first stage is completed. From the last or desquamating stage, in which the epidermis falls off, as far as the area reaches, in mealy or branny scales, the second characteristic of herpes is deduced.

### *Division and Description of the Species.*

### I. Division. Vesicular.

By vesicular herpes I understand those kinds, in which the *area* is somewhat redder, less elevated, and of greater circumference; and which, in the first stage, do not break out in pimples, or papulæ, but in vesicles, phlyctenæ. From the violent heat and itching, the vesicles are commonly broken by scratching on their first appearance; and then an an acrid lymph oozes out from their summits, which in the second stage forms a small yellow crust; so that the whole surface of the area becomes rough as a grater, or like the cutis anserina. As soon as the vesicles have poured out their contents, the cuticle forms rugæ in the whole extent of the area, and falls off in branny scales, which are larger than those which occur in the *papular* species.

I have hitherto had an opportunity of observing only three species of *vesicular* herpes. 1. The large transparent bladder-herpes, glass or porcelain-herpes, (herpes phlyctænoides. 2. The erysipelatous or confluent herpes, (h. erysipelatosus.) And 3. The miliary herpes, (h. miliaris.) These three kinds are sufficiently distinguished from each other by their external appearance, as well as by their intrinsic character, causes, and method of cure; but agree in the characteristics which have been pointed out. Area purpureo-rubescens, vesiculis corymbosis efflorescens. Willan, from his classing the different species of herpes under his Fourth Order (Bulla Vesicula) seems to know no other kind but the vesicular; but in his descriptions we here and there find an instance of herpes under lichen; and in his plates, under strophulus.

A. *Herpes Phlyctænoides*, Porcelaine Herpes. (Fig. 1.) I have twice had an opportunity of observing this eruption in young men, who had changed their residence and mode of life. Once I observed it in a girl, where it appeared as a critical excretion of a suppressed skin disease, or as a metastical eruption. The last time it appeared in rather smaller vesicles, which were regularly circular, or hemispherical. The patient was sixteen years of age, and had been affected with itch about half a year before this eruption appeared. The itch was suppressed by frictions with mercurial ointment, *which had occasioned* a tertian fever, and a retention of the menses. The patient was carried to a public hospital, where she had taken remedies for three weeks when I saw her; these consisted at first of evacuants and antimonials, and, after the fever abated, of gentle emmenagogues and the warm bath. After she had used the bath about ten times, this herpetic eruption appeared, after a dull pain in the body, on the right lumbar region; and at the same time the menses returned. All internal uneasiness ceased, and the appetite became good. The herpes on the second day extended upwards as far as the sixth rib on the right side, and under the right breast.

The

The areas were in general not smaller than a sixpence, and not larger than half-a-crown, very much inflamed and itching: none of them more distant from each other than a finger's breadth, and it was not more than half a day before vesicles were formed. The vesicles stood close to each other; most of them were of the size of a lentil, and reddish; at top white, shining, and pointed: few of them equalled a small pea in size. On the following day the vesicles had become glassy, pearl-coloured, and transparent; on the third day, those which had not been scratched nor burst, became wrinkled and collapsed; from some of them there oozed a viscid lymph, which formed a yellow crusty top. The epidermis of the area, which, in the inflammatory period, was elevated above the surrounding skin, became wrinkled about the fifth day, turned pale, and on the eighth fell off in branny scales, and sometimes even in large leaves. The area continued red, and appeared as if covered with white meal, and was circumscribed with a sharp margin: it itched five or six days longer, whilst the redness continued. When the lowest patch, near the mons veneris, was healed, the superior ones beneath the breast were in a state of inflammation. No application was made to the eruption except a little fresh expressed oil, which the patient was allowed to apply, to allay the itching.

The herpes of the two young men was much milder; but filled with larger and more irregular vesicles. They were both students from the country. It is supposed, and not unjustly, that most foreigners who drink the water here in Leipsic, get the itch or some other eruption; it happened to me, and to most of my countrymen: but perhaps the sedentary habits, mode of life, &c. of students, may have contributed towards it.

The first of these young men was a Swiss, aged 24, of a melancholico-phlegmatic temperament and a lax fibre. He had previously been accustomed to air, exercise, and wine daily; here he drank water, ate a quantity of truffles, cheese, and smoked flesh; was sedentery and studious. The eruption appeared near the lower part of the spine, and consisted of two inflamed areas of a purple red colour, between three and four inches long, and from one to two inches broad. These were thickly covered with large and small irregularly formed vesicles, which itched and burned so violently, that the patient could not bear his clothes to touch them. The vesicles were from the size of a millet seed to that of a barley corn or small bean; they

2 were were often curved obtusely triangular, and confluent; but had the glassy appearance, and resembled in their origin, progress, and other appearances, those of the girl. My patient was ordered to change his mode of living, to use gentle exercise in the open air, by which means the complaint was removed in three weeks.

The second person who suffered from this complaint was a law-student from Mühlhausen, a very industrious and regular young man of twenty years of age. He had had symptoms of dyspepsia; sat close in his study; but, except drinking water constantly, had committed no errors in diet. I ordered him the solnble tartar and fumaria, alternated with gentle laxatives, regular exercise, and free air. After a short journey on foot all his dyspeptic complaints disappeared. But this vesicular herpes arose in the left hypochondriac region: in eight days it had scarcely increased above three inches, and disappeared without either internal or external remedies: The Fig. I. was drawn from this eruption, which exactly agreed in every point with the two former. The specific distinction of this from other herpetic species, is its large transparent vesicles, with a true glassy lustre (glas-glanz), and resembling porcelain knobs. It ranks among the mild kinds of herpes. Retz, who refers all skin diseases to a plethora biliosa, has also given this a place.

B. *Herpes Erysipelatosus* — Fr. Dartre Erysipelateuse. H. pustulosus of Plenck. Sauvages. Spec. 4. (See Fig. II.) This species is distinguished from the former by its broader area diffused over a larger surface, and consisting of a cinnabar-coloured, burning, erysipelatous inflammation; which disappears gradually; is not circumscribed by a defined margin, and extends far beyond the few vesicles which are commonly placed in the middle of the area. The vesicles are preceded two or three days by the area, in the form of an itching, hot, erysipelatous patch. The largest vesicles occupy the central part, and commonly contain a yellowish acrid lymph; the smaller ones stand nearer the margin, and sometimes contain a purulent matter, and have a yellowish red point on their summits. The vesicles are smaller than in the former species, less elevated, and less transparent; have more of a *fatty* appearance, are less regular, and not so much crowded together. The smaller marginal vesicles form small crusts upon the apices, which makes the area in the second stage rough. Eschars or large crusts, as some pretend, I have never observed; served; the larger central vesicles form no crusts on their tops, but become pale and fall into wrinkles until the elevated cuticle dries and falls off in small patches. This species of herpes is of the moist kind, slower and more obstinate in its progress than the former, but by no means so malignant as to have it classed with Sauvages (Spec. 4.) among the spreading kind (H. estromenos of Galen. H. exedens vel depascens of Turner. Spec. 4). It is distinguished from the erysipelas bullosum by its area, which the last wants, and by the congregated small bullæ, which in the erysipelas are larger and more distinct. It is also much more itchy than the pure erysipelas, which never terminates by a branny desquamation. Fever never accompanies it, as sometimes happens in the erysip. bullosum. It is commonly seated upon the exposed parts of the breast and arms, often also upon the neck. It is peculiar to females.

In private practice this eruption has occurred to me only once, when I treated it merely internally. It was of the benign kind, and radically cured in 12 days. The patient was a young woman, who had been frequently troubled with erysipelas in the face, and since her childhood had been subject to lichenous eruptions on her neck and chin. She had now been much heated by washing, and, whilst perspiring, suddenly exposed herself to a piercing north wind in January; this eruption immediately appeared in her neck; in three days it proceeded as far as her right ear, and formed six distinct red herpetic spots, which touched each other, and threw out the above described bullæ in their centres. Two, of these patches were extremely painful, and by the constant oozing of a sharp corrosive lymph, became excoriated and bloody; One of these is represented at B. (See plate, fig. 2.)

I have seen this species three times in hospitals, where it was always more tedious and obstinate. Upon the foot of a woman thirty-two years of age, it had continued three months; the patient had been treated, as I was informed, with fumitory, sarsaparilla, dulcamara, cicuta, antimony, and corrosive sublimate. In one case, the internal and external use of vitriolic acid was said to have been useful. My patient took every morning a gentle aperient, after which she drank an infusion of the flowers of verbascum. At night, when warm in bed, she took about 60 drops of liver of sulphur in a cup of a gentle diaphoretic infusion. In order to sooth the burning itching pain, she was allowed to apply externally only the ungt. de uvis.

C. *Herpes*

C. *Herpes Miliaris*, Sennert. lib. 5. c. 17.—Dartre miliare, Poupart, vid. Plenck, spec. 5. Turner, Sauvages. (Plate, fig. 3.) This is the most malignant and obstinate of all the vesicular species: it is not so rare as the two former species, and attacks men, women, and children; it affects the arms and legs, neck, head, breast, and abdomen. It appears at first in an insignificant form, viz. in small vesicles like millet seeds, of a pale red colour, and pointed, with very pale apices (A. Fig. III). These are situated upon an elevated area, moderately inflamed when compared with the erysipelatous herpes, but itching violently. In the second stage the vesicles become more crowded, and the burning and itching of the area increase, (B). The pimples or vesicles soon burst, or are broken by scratching; a saline glutinous fluid oozes out, which increases the redness and heat; the cuticle is eroded by it, and the herpes spreads. When the inflammation of the area is violent, the vesicles are sometimes filled with a purulent matter, which has a yellow appearance seen through the transparent tips of the vesicles; but I have also seen them watery, and quite empty, hard and almost papulous. I have never observed fever in this complaint. When it has existed for a length of time, the inflamed surfaces touch each other, and become confluent, so that a single area is no longer to be distinguished. The whole surface or basis is red, becomes eroded in the middle, and excoriated by the constant oozing out of an acrid lymph; it is also full of bloody chinks or furrows, surrounded by a fresh margin of miliary eruptions. Of this I have given a representation in Fig. III. C.

No kind of herpes in general bears to be covered without uneasiness; the dry kinds are irritated by the friction of the shirt and clothes, and the itching is thus increased. Heat also produces the same effect. The moist kinds are likewise troublesome, by sticking to the linen as soon as they burst, which being again torn away on using motion, the cutis is left naked. This is especially the case with the H. miliaris, in which the rete mucosum is thus laid bare, and the small vessels of the skin, being torn, pour out blood. To prevent this, the eruption should be anointed with simple ointment, which will also assuage the pungent itching. Some physicians imagine that this species is communicated by infection, e. g. by means of linen, barber's razors, towels, &c. I cannot speak from experience on this point. It is certain, however, that this kind of herpes is for the most part accompanied by evident marks marks of depraved fluids, which gives the physician much trouble; for when he supposes the eruption removed, it returns again, and fresh vesicles often spring up on an area which has already had two successive crops. This is particularly the case in scrophulous and scorbutic subjects. This kind of herpes does not appear to be hereditary: it arises frequently after a suppression of the hæmorrhoidal or menstrual discharges. In general it shews a striking connection with the periodical discharge in women; e. g. it appears only at the beginning of this period, or disappears during that time, or on its cessation from age; or it vanishes during the period of pregnancy, and re-appears after delivery. Dirty people, and natives of warm countries who eat much oil and fish, are peculiarly liable to this eruption. It occurs particularly in moist and marshy countries, in spring and autumn. Many internal diseases excite it; and many others arise in consequence of its being suppressed by cold, frictions with mercurial or saturnine ointment, &c.

The remedies to be employed for the cure of this eruption must be different, according to the causes which have produced it. If it be occasioned by a cachectic state of the fluids, it must be remedied by a change in the mode of living, and by medicines which purify the blood. If it arise from metastasis, it must be healed with liver of sulphur, sulph. aurat. antimony, warm bathing, and evacuants. If it be connected with suppression of the hæmorrhoids, menses, &c. these discharges must first be restored. Some consider a metastasis of gouty matter as a cause of herpes: others, that it is a peculiar herpetic acrimony, for which corros. sublimate is the antidote. In hospitals I have once seen this eruption treated according to Smyth's plan, internally and externally with cantharides: another time according to Thilenius, internally with dulcamara, rad. lapathi, and fumaria, and externally with elm bark. Constantini has speedily cured it with a salve composed of litharge, lap. calamin. ol. oliv. and acetum, with which the area was covered twice a day, at the same time the perspiration was kept up by a warm infusion of elder flowers; others have had success with belladonna and rhubarb. The Portuguese cure it by the external use of lemon juice, and the leaves and flower-cups of the cistus ladaniferus; and internally with a powder of the burnt ossa sepiæ, calomel, and crude antimony, whilst at the same time caldas, or sulphur water, or aqua de S. Miguel is drank.*

* The *papulous* herpes, together with an account of the general mode of cure, will appear in a future number of the Paradoxieu.

## Figures and Tables

**Fig.1. Fig. 2.A Fig. 2.B Fig. 3.A Fig. 3.B Fig. 3.C f1:**