# Errata

**Published:** 1804-04-01

**Authors:** 


### ERRATA.

Page 230, line 26, *for* designing, *read* designating.
laſt line, after strophulus, dele comma.

| | | | |
|---|---|---|---|
| 232, | line 24, *for* | papulæ, *read* | papula. |
| | 36, | imitates, | irritates. |
| 233, | 1, | fonthi, | ionthi, |
| 234, | 2, 3, | papilla, | papillæ. |
| 235, | 28, | metastical, | metastatical. |
| 240, | 25, | antimony, | antimonii. |

